# Assisted reproductive technology pregnancy complications are significantly associated with endometriosis severity before conception: a retrospective cohort study

**DOI:** 10.1186/s12958-016-0209-2

**Published:** 2016-11-03

**Authors:** Tatsuya Fujii, Osamu Wada-Hiraike, Takeshi Nagamatsu, Miyuki Harada, Tetsuya Hirata, Kaori Koga, Tomoyuki Fujii, Yutaka Osuga

**Affiliations:** Department of Obstetrics and Gynecology, University of Tokyo, 7-3-1 Hongo, Bunkyo-ku, Tokyo, 113 8655 Japan

**Keywords:** Endometriosis, Preterm birth, Placenta previa, Revised American Society for Reproductive Medicine Stage

## Abstract

**Background:**

Endometriosis has been shown to be associated with second- to third-trimester pregnancy complications such as preterm birth and placenta previa, but the evidence is inconsistent. We hypothesized that endometriosis severity might affect these inconsistent results. Therefore we aimed to conduct a retrospective cohort study to elucidate whether endometriosis severity is associated with the incidence rates of adverse pregnancy outcomes.

**Methods:**

The patients who achieved singleton pregnancy by assisted reproductive technology (ART) in our facility between March 2000 and December 2014 (*N* = 631) were included in this analysis. Among them, 92 women demonstrated surgically proven endometriosis, and 512 women were shown to not have endometriosis as a complication. Among the 92 cases of endometriosis, 10 were classified as revised American Society for Reproductive Medicine (rASRM) stage I and II, 31 cases were rASRM stage III, and 43 cases were rASRM stage IV; in 8 cases, the rASRM stage was unavailable. Logistic regression analysis was performed to calculate odds ratios (OR) and 95 % confidence interval (CI) for the rates of preterm birth, placenta previa, and small for gestational age. OR were adjusted by age, parity and the number of transferred embryos.

**Results:**

First we confirmed the frequency of preterm birth and placenta previa were significantly increased in women with endometriosis (preterm birth OR, 2.08; 95 % CI, 1.07–3.89, placenta previa OR, 15.1; 95 % CI, 4.40–61.7), while the frequency of small for gestational age was not. Moreover, we found the frequencies of preterm birth and placenta previa were significantly increased in women with rASRM stage IV endometriosis compared to other two groups: women with rASRM stage I-III endometriosis (preterm birth OR, 7.40; 95 % CI, 1.83–50.3; placenta previa OR, 11.0; 95 % CI, 1.75–216.5) and women without endometriosis (preterm birth adjusted OR, 4.11; 95 % CI, 1.88–8.55; placenta previa adjusted OR, 39.8; 95 % CI, 10.1–189.1). There were no significant difference between women with rASRM I-III endometriosis and women without endometriosis.

**Conclusions:**

We found that the frequencies of preterm birth and placenta previa were significantly increased in women with endometriosis, and the severity of endometriosis might have an adverse impact on ART pregnancy.

## Background

Endometriosis is defined as an abnormal development of endometrial tissue outside the uterus. This condition causes chronic inflammation in the pelvis of reproductive-aged women. Estrogen is associated with the pathogenesis of endometriosis because the symptoms related to endometriosis resolve soon after the cessation of menstruation. The chronic inflammation results in the generation of ovarian endometrioma and the formation of adhesions in the reproductive organs; thus, it is well known that this disease is associated with infertility [1, 2]. In addition, the T helper (Th)1/Th2 balance has been established to be disrupted in patients with endometriosis [3]. Moreover, endometriosis might affect the expression patterns of progesterone receptors (PR) A and PR B [4].

Recently, endometriosis has been shown to be associated with second- to third-trimester pregnancy complications, such as preterm birth, preeclampsia, small for gestational age (SGA) and placenta previa [3–6]. However, several investigators have reported that the association between endometriosis and pregnancy complications is questionable [7–9], and the association remains under debate [10, 11]. Some reports have suggested that not endometriosis itself but ovarian endometrioma and rectovaginal endometriosis might be associated with adverse pregnancy outcomes. A retrospective cohort study suggested that ovarian endometrioma was associated with preterm birth, but endometriosis without ovarian endometrioma was not associated with preterm birth in assisted reproductive technology (ART) pregnancy [12]. An association between placenta previa and rectovaginal endometriosis has been previously reported [7]. However multivariate analysis was not performed in this study, and evidence is limited. From these observations, it is still difficult to conclude what types of endometriosis increases the risk for pregnancy complications.

In general, minimal and mild endometriosis correspond to peritoneal disease, while moderate endometriosis corresponds to one ovarian endometrioma > 3 cm, and severe endometriosis corresponds to bilateral endometriomas and/or complete pouch of Douglas obliteration, according to the revised American Society for Reproductive Medicine (rASRM) scoring system, which is widely used to evaluate and stage endometriosis severity [1]. To take into account the association between ovarian endometrioma or rectovaginal endometriosis and the increased rates of preterm birth and placenta previa, we hypothesized that rASRM stage might affect the incidence rates of these pregnancy complications. Therefore, the aim of this study was to evaluate the association between endometriosis and pregnancy complications as well as to investigate the influence of endometriosis severity on pregnancy complication outcomes using the rASRM scoring system.

## Methods

### Sample collection

The demographic data of each case were recorded in a medical record of our hospital. These data included the patient’s age, parity, past medical history including surgical records, suspected causes of infertility, the number of transferred embryos and pregnancy outcomes. Sample collection was performed by extracting data from these records. According to the database, 631 singleton babies were born as a result of ART performed in our hospital between March 2000 and December 2014.

### Diagnosis and surgical treatment of endometriosis

Laparoscopy is the most reliable procedure for diagnosing and classifying endometriosis [1]. Therefore, current data were based on laparoscopic findings to accurately evaluate the risks of pregnancy with endometriosis. In each case, therapeutic procedures, such as adhesiotomy, coagulation, and cystectomy, were performed by trained personnel at our hospital to treat the detected endometriosis.

**Fig. 1:**
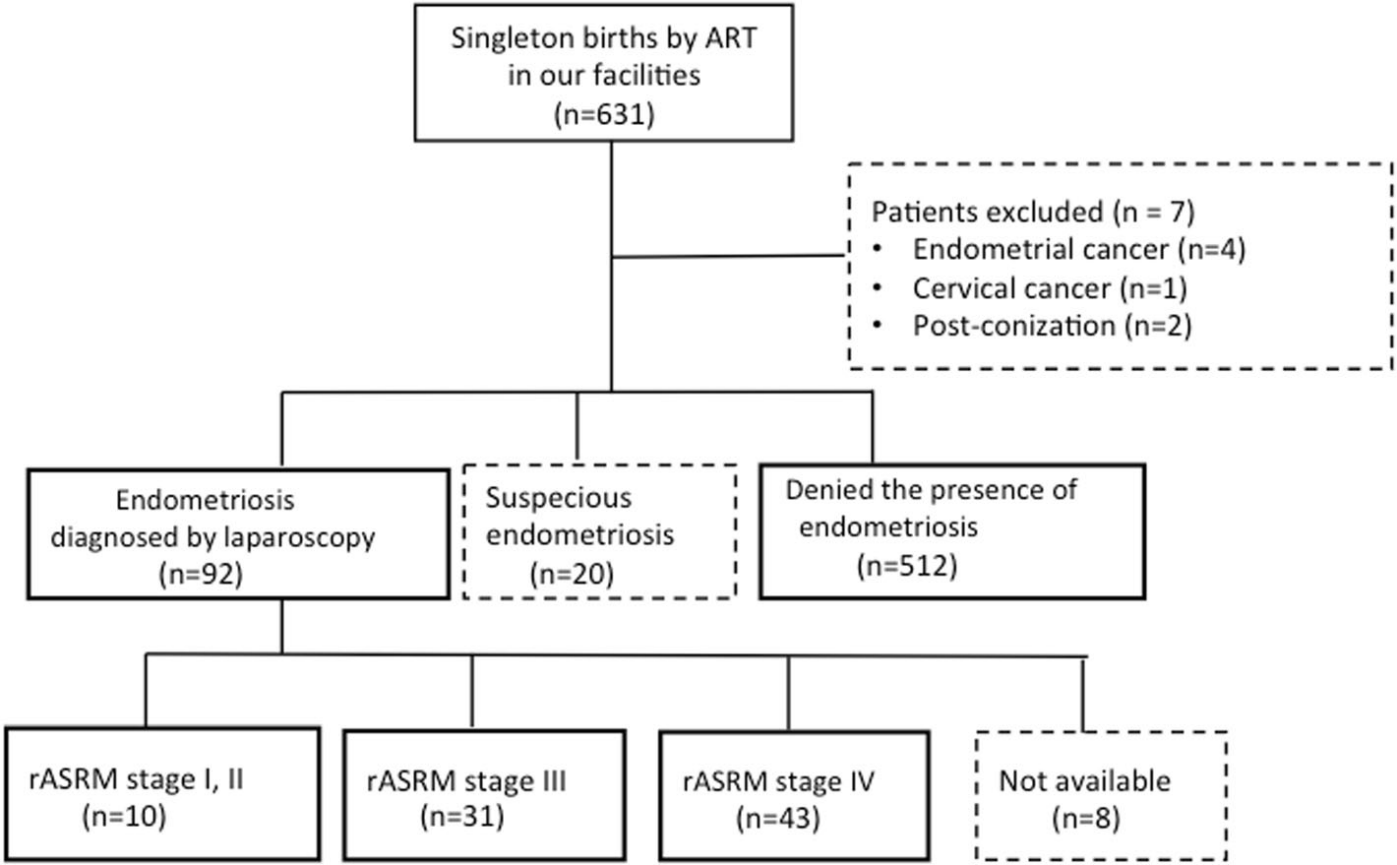
Flowchart of sample collection criteria. Among 631 cases, 7 cases were excluded because of the following complications: past medical history for the treatment of endometrial cancer (*n* = 4), cervical cancer diagnosed in early pregnancy (*n* = 1), and previous conization (*n* = 2). Of the remaining 624 cases, laparoscopically diagnosed endometriosis was present in 92 cases, which were classified as “endometriosis diagnosed by laparoscopy.” Twenty cases were excluded from this study because endometriosis was suspected but not diagnosed by laparoscopy. The other 512 cases were classified as “denied presence of endometriosis.” Among the 92 cases of “endometriosis diagnosed by laparoscopy,” 10 cases were classified as rASRM stage I or II, 31 cases were rASRM stage III, and 43 cases were rASRM stage IV. In eight cases, the rASRM stage was unavailable. ART: assisted reproductive technology, rASRM: revised American Society for Reproductive Medicine

The women who had laparoscopically diagnosed endometriosis were categorized as “endometriosis diagnosed by laparoscopy” (*n* = 92). The women who were suspected to have endometriosis by transvaginal ultrasound or medical history but did not undergo laparoscopy were categorized as having “suspected endometriosis” and were excluded from this study (*n* = 20). The other women who were not suspected to have endometriosis by transvaginal ultrasound or in whom the presence of endometriosis was denied by laparoscopy were categorized as “denied presence of endometriosis” (*n* = 512). The women classified as “endometriosis diagnosed by laparoscopy” and “denied presence of endometriosis” were included in this study. Seven women were excluded because of complications before pregnancy; four suffered endometrial cancer, one was diagnosed with cervical cancer in early pregnancy, and two were recovering from conization. We used the rASRM scoring system to evaluate the severity of endometriosis because this is a widely accepted scoring system [1]. Of the 92 cases of laparoscopically diagnosed endometriosis, 10 cases were rASRM stage I and II, 31 were rASRM stage III, and 43 were rASRM stage IV; in 8 cases, the rASRM stage was unavailable. Sample collection criteria was summarized in Fig. 1.

### Complication outcomes during pregnancy

We analyzed following adverse pregnancy outcomes: preterm birth, placenta previa, and SGA. The definitions used in this study for the diagnosis of these complications was as follows. Preterm birth was defined as delivery before 37 complete weeks of gestation. Placenta previa was defined as the condition in which the placenta at least partially covered the internal ostium. SGA defined as an infant weight less than the 10^th^ percentile for the gestational age.

### Statistical analysis

All statistical analyses were performed using JMP PRO 11 software (SAS Institute Inc., Cary, NC, USA). Fisher’s exact test was used to calculate *p* values for the comparison of maternal characteristics with or without endometriosis. Logistic regression analysis was used to calculate the adjusted odds ratios (AORs) and 95 % confidence intervals (CIs) in the analysis of adverse pregnancy outcomes. Odds ratios were adjusted by age, parity and the number of transferred embryos.

## Results

### Characteristics of the patients included in this study

The preconceptional characteristics of the patients included in this study are summarized in Table 1. There were no significant differences in age, previous parity or the number of embryos transferred between the women with or without endometriosis.

**Table 1:** Characteristics of women with ART pregnancy

	ART with endometriosis	ART without endometriosis	*P* value
*N*	92	512	
Age (year, mean)	34.7	35.4	0.078
Previous parity			0.29
0	78 (84.8 %)	396 (77.6 %)	
1	14 (15.2 %)	113 (22.2 %)	
2	0	1 (0.2 %)	
No. of ET for conception			0.788
1	55 (59.8 %)	292 (57.0 %)	
2	28 (30.4 %)	151 (29.5 %)	
3	9 (9.8 %)	68 (13.3 %)	
4	0	1 (0.2 %)	

Note: *ART* assisted reproductive technology; *ET* embryos transferred

### Preterm birth and placenta previa were increased in women with endometriosis

The risks of preterm birth, placenta previa, and SGA in ART pregnancy with or without endometriosis are shown in Table 2. The risks of preterm birth and placenta previa were significantly increased in the pregnant women with endometriosis (preterm birth AOR, 2.08; 95 % CI, 1.07–3.89, placenta previa AOR, 15.1; 95 % CI, 4.40–61.7). No significant difference was found in the risk of SGA.

**Table 2:** Complications during pregnancy in women with or without endometriosis

	ART with endometriosis	ART without endometriosis	Odds ratio (95 % Cl)^a^	*P* value
Preterm birth	15 (16.5 %)	43 (8.5 %)	2.08 (1.07–3.89)	0.032
Placenta previa	8 (9.1 %)	4 (0.8 %)	15.1 (4.40–61.7)	< 0.001
SGA	11 (14.1 %)	52 (10.3 %)	1.43 (0.68–2.81)	0.332

*SGA* small for gestational age, *CI* confidence interval

^a^Adjusted by age, parity, and the number of transferred embryos

### Preterm birth and placenta previa were increased in women with rASRM stage IV but not stage I-III endometriosis

The risks of adverse pregnancy outcomes were compared among the following three groups: the women with rASRM stage I–III endometriosis, the women with rASRM stage IV endometriosis and the women without endometriosis. The risks of preterm birth and placenta previa were significantly increased in the women with rASRM stage IV endometriosis compared to the women with rASRM stage I-III endometriosis (preterm birth AOR, 7.40; 95 % CI, 1.83–50.3; placenta previa AOR, 11.0; 95 % CI, 1.75–216.5) and to the women without endometriosis (preterm birth AOR, 4.11; 95 % CI, 1.88–8.55; placenta previa AOR, 39.8; 95 % CI, 10.1–189.1). However, the risks of these adverse pregnancy outcomes were not increased in the women with rASRM stage I-III endometriosis compared to the women without endometriosis. No significant differences were found in the risk of SGA between any two groups (Table 3).

**Table 3:** Comparison of complications classified by endometriosis severity

	rASRM stage I – III	Odds ratio (95 % Cl)^a^	rASRM stage IV	Odds ratio (95 % Cl)^a^	Odds ratio (95 % Cl)^b^
Preterm birth	2 (4.9 %)	0.55 (0.09–1.90)	12 (27.9 %)	4.11 (1.88–8.55)***	7.40 (1.83–50.3)***
Placenta Previa	1 (2.4 %)	3.61 (0.18–26.3)	7 (17.1 %)	39.8 (10.1–189.1)***	11.0 (1.75–216.5)***
SGA	4 (10.3 %)	0.99 (0.29–2.61)	7 (20.6 %)	2.30 (0.88–5.37)	2.32 (0.63–9.70)

*rASRM* revised American Society for Reproductive Medicine

***p < 0.05

^a^Adjusted odds ratio toward ART without endometriosis

^b^Adjusted odds ratio of rASRM stage IV toward rASRM stage I-III

## Discussion

From this study, we found that ART pregnancy complicated with endometriosis carries a high risk of preterm birth and placenta previa, especially in women with rASRM stage IV endometriosis. The incidence rates of preterm birth and placenta previa in rASRM stage IV (27.9 and 17.1 %, respectively) were surprisingly high compared to the general incidence rates reported in Japan (5–6 and 0.5–0.7 %, respectively) [12, 13]. These data suggest that there is a strong association between severe endometriosis and these pregnancy complications. To our knowledge, this is the first report to suggest the correlation of endometriosis severity and adverse outcomes in the second and third trimester of pregnancy.

This study was only included ART pregnancies to avoid the influence of conception methods. ART pregnancy is known to carry a high risk of adverse pregnancy outcomes, such as preterm birth and placenta previa, compared with natural conception [14, 15]; however, the underlying mechanism of the increased risk for adverse outcomes in ART pregnancy remains unclear. One report suggests that not only the ART procedure itself but also underlying causes of infertility might be associated with adverse pregnancy outcomes in ART pregnancy [16]. According to this point of view, we could also conclude that endometriosis, one of the most prevalent diseases causing infertility in women, might be a crucial factor of increased adverse outcomes in ART pregnancy.

On the other hand, rASRM stage I-III endometriosis followed by therapeutic laparoscopic procedures might not be associated with second- and third- trimester pregnancy complications. While the sample size of rASRM stage I-III endometriosis in this study was insufficient to completely deny the association between minimal to moderate endometriosis and pregnancy complications, at least strong associations were not recognized in this study. These results seemed to be inconsistent with those of past studies, which suggested an association of endometrioma with preterm birth [12]; however, we have to consider that no endometriomas were present at the time of conception in most cases included in this study because of therapeutic procedures performed for endometriosis. In that context, our results are consistent with those of past studies.

These results are also interesting in terms of the mechanisms underlying the increased risks of pregnancy complications in women with endometriosis. In most cases in this study, ovarian endometriomas were almost completely removed by laparoscopic surgery, and apparent recurrences were not observed. However, strong adhesions mainly recognized in rASRM stage IV cases were not completely removed. We speculate that these remaining adhesions and inflammation in the rectovaginal region might be the main cause of the increased risks of preterm birth and placenta previa, as previously suggested [7].

This study also had some limitations. Adenomyosis is a disease characterized by the presence of endometrial tissue deep within the uterine myometrium [17], and an association of adenomyosis with preterm birth has been previously reported [16, 17]. Therefore, adenomyosis might have influenced the increased incidence rate of preterm birth in this study. On the other hand, to the best of our knowledge, no reports have suggested an association between adenomyosis and placenta previa. Further research is needed to elucidate the influence of adenomyosis on the increased risk of preterm birth in women with endometriosis.

## Conclusion

We found that ART pregnancies complicated with endometriosis, especially laparoscopically diagnosed severe endometriosis, and carried a high risk for preterm birth and placenta previa. These findings are clinically relevant to obstetricians for distinguishing high- and low-risk pregnancies.

## Abbreviations

AOR: Adjusted odds ratio
ART: Assisted reproductive technology
CI: Confidence interval
PR: Progesterone receptor
rASRM: Revised American Society for Reproductive Medicine
SGA: Small for gestational age
Th: T helper
